# Serum miR-17, IL-4, and IL-6 levels for diagnosis of endometriosis

**DOI:** 10.1097/MD.0000000000010853

**Published:** 2018-06-15

**Authors:** Fang Wang, Hongxia Wang, Danting Jin, Yang Zhang

**Affiliations:** a Department of Gynecology; b Department of Pathology; c Department of Clinical Laboratory, The First People's Hospital of Lianyungang, Xuzhou Medical University Affiliated Hospital of Lianyungang, Lianyungang, Jiangsu, China.

**Keywords:** endometriosis, IL-4, IL-6, microRNA, miR-17

## Abstract

Clinical studies have exhibited microRNAs or cytokines could be used as new biomarkers in the diagnosis of endometriosis, respectively. The purpose of this study was to investigate the role of serum miR-17, IL-4, and IL-6 as early diagnostic markers of endometriosis. One hundred forty patients aged 22 to 45 years were recruited, 80 patients with pathologically confirmed endometriosis were assigned to endometriosis group whereas the remaining 60 patients were in the control group. The blood samples were collected immediately before laparoscopy and analyzed using real-time quantitative PCR analysis. In patients with endometriosis, the level of miR-23b decreased significantly, the levels of IL-4 and IL-6 increased remarkably compared with that in patients without endometriosis. Correlation analysis revealed miR-17 levels were negatively correlated with IL-4 (*r* = −0.974, *P* < .05) and IL-6 (*r* = −0.944, *P* < .05). The ROC curve manifested joint of miR-17 and selected cytokines could improve the diagnostic power with an AUC of 0.84 (95% CI: 0.75–0.96). In short, the present study characterizes the role of miR-17, IL-4, and IL-6 in the pathogenesis of endometriosis, suggesting the feasibility of using miR-17 and selected cytokines as a noninvasive diagnostic test for the detection of endometriosis.

## 1 Introduction

Endometriosis is one of the most common gynecological diseases, in which active endometrial cells are planted outside of the endometrium.^[1]^ The lesions can affect all pelvic tissues and organs such as ovaries, rectal fovea, the sacrosacral ligament, and the pelvic peritoneum.^[2]^ Symptoms include dysmenorrhea, dyspareunia, dyschezia, dysuria, chronic pelvic pain, and infertility.^[3]^ Laparoscopic visual examination and pathological diagnosis are the gold standard for the diagnosis of endometriosis.^[4]^ However, previous studies have revealed that these examinations could show a considerable delay in diagnosing endometriosis which may worsen the endometriosis lesions.^[5,6]^

MicroRNAs are a class of highly conservative noncoding small RNAs (approximately 21–24 nucleotides) that regulate gene expression in signal pathways, which play an important role in maintaining the homeostasis.^[7]^ It regulates posttranscriptional gene silencing, promotes the mRNA degradation or inhibits the transcription by recognizing the 3′-UTR of target mRNA.^[8]^ The differential expression of identified microRNAs in plasma of women with endometriosis implied that microRNAs likely act as potent participants in the progression of endometriosis.^[9,10]^ Jia et al^[11]^ have found that expression of miR-17 was reduced in women with endometriosis, which may be a basis for the diagnosis of endometriosis.

Endometriosis is considered to be an inflammatory process in which activated immune-related cells secreted large amounts of cytokines.^[12]^ These cytokines could attract more immune cells and promote growth of ectopic endometrial cells, contributing to the occurrence and development of endometriosis.^[13,14]^ Previous studies have indicated a link between raised serum levels of IL-4 or IL-6 and endometriosis.^[15,16]^

So far, clinical studies have exhibited microRNAs or cytokines could be used as new biomarkers in the diagnosis of endometriosis. There is limited data about joint detection of microRNAs and cytokines in plasma of patients with endometriosis. Accordingly, we aim to investigate the role of serum miR-17, IL-4, and IL-6 as early diagnostic markers of endometriosis.

## 2 Materials and methods

### 2.1 Patient enrollment

One hundred forty patients aged 22 to 45 years were recruited from January 2016 to January 2017 in The First People's Hospital of Lianyungang. All patients in this study received laparoscopy on account of gynecological indications such as suspected endometriosis, pelvic masses, pelvic pain, infertility, and uterine leiomyoma. Of 140 patients, 80 patients with pathologically confirmed endometriosis were assigned to endometriosis group whereas the remaining 60 patients were in the control group. The extent of endometriosis was classified in accordance with the American Society of Reproductive Medicine (ASRM) revised system^[17]^: stage I (n = 22), stage II (n = 28), stage III (n = 20), and stage IV (n = 10). And no pelvic pathologies (n = 5), simple ovarian cysts (n = 12), uterine leiomyoma (n = 16), and unexplained infertility (n = 7) were detected in the control group. This study was approved by the ethics committee of The First People's Hospital of Lianyungang, and written informed consents were obtained from all participants.

### 2.2 Samples collection

The blood samples were collected by peripheral vein puncture immediately before laparoscopy. Whole blood was centrifuged at 1200*g* for 15 minutes within 30 minutes of collection, followed by plasma separation which was frozen at −80°C as described.^[11]^

### 2.3 Real-time quantitative PCR analysis

The total RNA was extracted from the plasma using TRIzol reagent (Invitrogen, Carlsbad, CA) according to the manufacturer's protocols. Complementary DNA (cDNA) was synthesized using a reverse transcription kit (Takara Biotechnology, Dalian, China). Relative quantity of cDNA was analyzed by quantitative polymerase chain reaction (PCR) with SYBR green and the ΔΔCT method.^[18]^ All primers were designed and synthesized by GenePharma (Shanghai, China) and shown as follows: miR-17, CAAAGTGCTTACAGTGCAGGTAG; IL-4, AACGGCTCGACAGGAACCT and ACTCTGGTTGGCTTCCTTCCA; IL-6, GAGGATACCACTCCCAACAGACC and AAGTGCATCATCGTTGTTCATACA; β-actin, CACGATGGAGGGGCCGGACTCATC and TAAAGACCTCTATGCCAACACAGT. Relative levels of gene expression was expressed relative to β-actin and calculated using the 2^−ΔΔCt^ method.

### 2.4 Statistical analysis

All calculations were performed using SPSS17.0 software and GraphPad, the normal distribution measurement data was measured as the mean ± SD, and the *t* test was used for comparison between the variable groups. Categorical variables were compared using Chi-square test. The correlation analysis of continuous variables was based on Spearman test correlation method. The diagnostic efficacy of the plasma miR-17 and selected cytokines was analyzed with the receiver operating characteristic (ROC) curve, and the area under the ROC curve (AUC) was calculated. A value of *P* < .05 indicated a statistically significant difference.

## 3 Result

### 3.1 Serum miR-17, IL-4, and IL-6 level in patients with endometriosis

**Figure 1:**
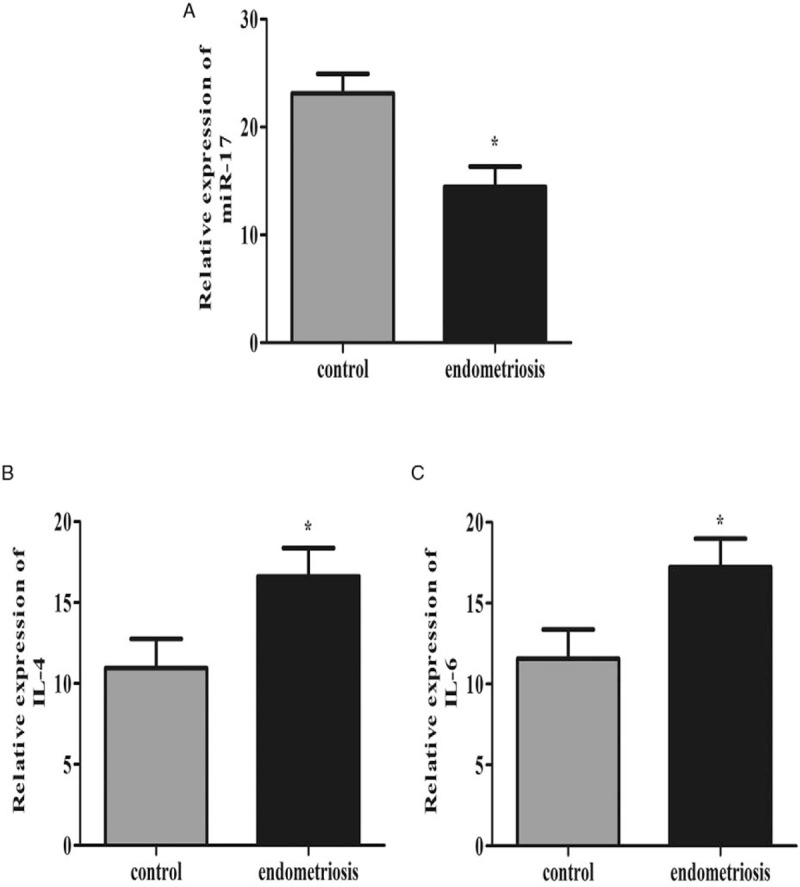
Serum miR-17, IL-4, and IL-6 level in patients with endometriosis. miR-23b expression level in AMI and healthy controls. (A) Plasma level of miR-17 in endometriosis decreased significantly compared with control group. (B) Plasma level of IL-4 in endometriosis was significantly elevated compared with control group. (C) Plasma level of IL-6 in endometriosis was significantly elevated compared with control group.

In order to explore the role of miR-17, IL-4, and IL-6 in the development of endometriosis, the expression of these indexes was analyzed using qPCR. As shown in Fig. 1A, the level of miR-17 was downregulated in endometriosis group significantly. Moreover, the IL-4 and IL-6 expression exhibited an increase in endometriosis compared to that in control group (*P* < .05; Fig. 1B and C).

### 3.2 Serum miR-17, IL-4, and IL-6 level in different grades of patients with endometriosis

The endometriosis patients were classified into 4 grades according to the ASRM revised system. And the level of miR-17, IL-4, and IL-6 was analyzed based on different grades in endometriosis group (Table 1). The results showed miR-22 expression decreased along with the higher endometriosis grade significantly whereas the level of IL-4 and IL-6 markedly decreased.

**Table 1:**
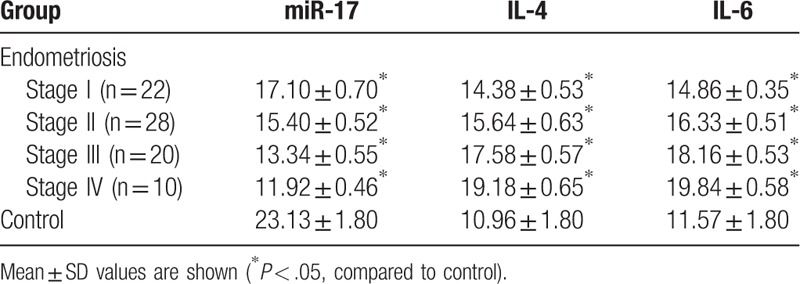
miRNA-17, IL-4, and IL-6 expressions levels in control and patients groups with r-AFS.

### 3.3 miR-17 targets with 3′-UTR of IL4 receptors and IL-6 receptors

**Figure 2:**
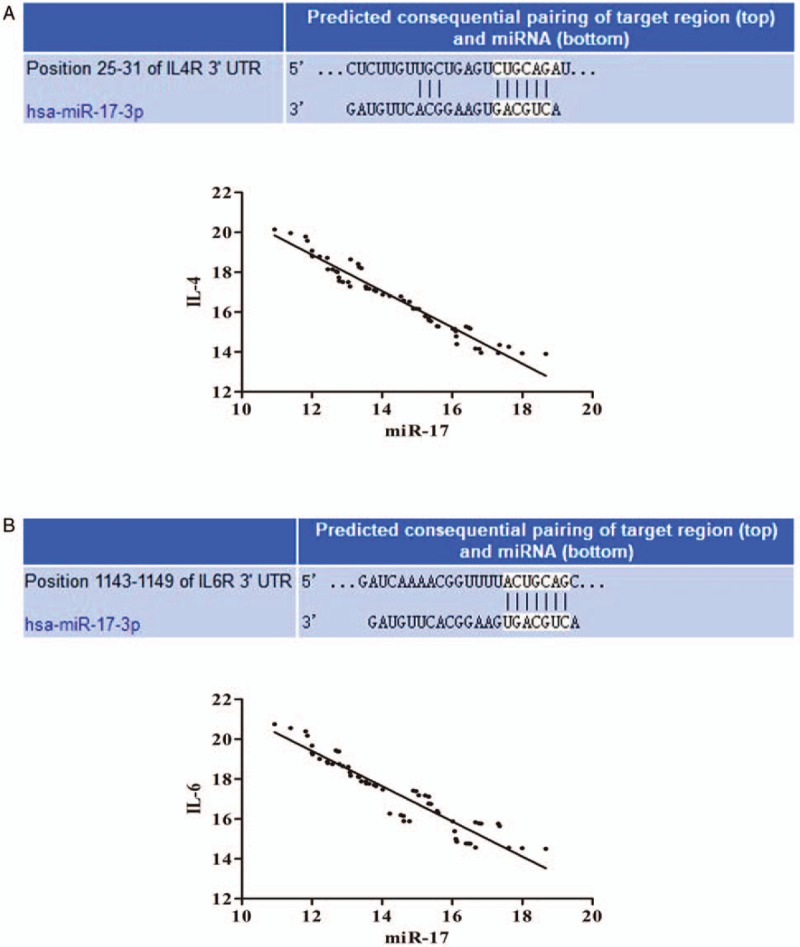
miR-17 targets with 3′-UTR of IL4 receptors and IL-6 receptors. miR-17 targeted with 3′-UTR of IL-4 receptor and IL-6 receptor in different positions closely. The 3′-UTR binding sites can be seen in (A) and (B). Correlation analysis revealed that miR-17 levels were negatively correlated with IL-4 (*r* = −0.974, *P* < .05; A) and IL-6 (*r* = −0.944, *P* < .05; B).

Bioinformatics analysis was used to screen the potential targeting miRNAs binding with miR-17. Results demonstrated that miR-17 targeted with 3′-UTR of IL-4 receptor and IL-6 receptor in different positions closely. The 3′-UTR binding sites can be seen in Fig. 2A and B. Furthermore, correlation analysis revealed that miR-17 levels were negatively correlated with IL-4 (*r* = −0.974, *P* < .05; Fig. 2A) and IL-6 (*r* = −0.944, *P* < .05; Fig. 2B).

### 3.4 ROC curve analysis using serum miR-17 and selected cytokines for discriminating endometriosis

**Figure 3:**
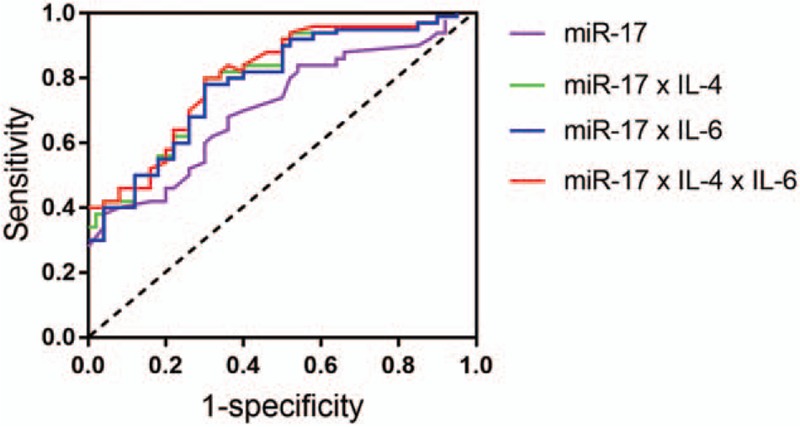
ROC curve analysis using serum miR-17 and selected cytokines for discriminating endometriosis. ROC curve analyses revealed that the plasma levels of miR-17 was potential biomarker for diagnosis of endometriosis with AUC values of 0.75 (95% CI: 0.55–0.86). When the 3 parameters were combined by multiplication (miR-17 × IL-4, miR-17 × IL-6, and miR-17 × IL-4 × IL-6, respectively), the AUC values were 0.79 (95% CI: 0.68–0.87), 0.81 (95% CI: 0.71–0.91), and 0.84 (95% CI: 0.75–0.96), respectively.

ROC curve analyses revealed that the plasma level of miR-17 was potential biomarkers for diagnosis of endometriosis with AUC values of 0.75 (95% CI: 0.55–0.86). We also observed joint of miR-17 and selected cytokines could improve the diagnostic power. When the three parameters were combined by multiplication (miR-17 × IL-4, miR-17 × IL-6, and miR-17 × IL-4 × IL-6, respectively), the AUC values were 0.79 (95% CI: 0.68–0.87), 0.81 (95% CI: 0.71–0.91), and 0.84 (95% CI: 0.75–0.96), respectively (Fig. 3).

## 4 Discussion

Blood is regarded as potential source of biomarkers because it is conveniently obtained with good repeatability.^[19]^ Many factors in plasma have been regarded as potential biomarkers for the early diagnosis of endometriosis, including glycoproteins, growth factors, hormones, or proteins related to immunology or angiogenesis.^[20,21]^

Circulating microRNAs were firstly identified as potential biomarkers serum from patients with diffuse large B cell lymphoma in 2008.^[22]^ Reduced plasma level of miR-17 had been found in women with endometriosis compared with women without endometriosis.^[11]^ However, all cases were the revised classification of the American Fertility Society (rAFS) stage III–IV, which may limit generalization of plasma microRNAs for early diagnosis of endometriosis. The present study showed miR-17 expression was lower in endometriosis group than that in control group. Besides, there was a significant decline of miR-17 in endometriosis with different stages.

Inflammatory factors have been implicated in the progression of endometriosis. Clinical studies have confirmed close relationship between cytokines and endometriosis, suggesting that cytokines could be used as predictors of this disease.^[21]^ For example, Drosdzol-Cop et al found adolescent patients with endometriosis displayed significantly higher serum IL-4,^[15]^ Othman Eel et al^[23]^ observed serum interleukin-6 measurements discriminate between women with endometriosis and without endometriosis. Consistent with these findings, the results showed the remarkable elevated concentration of IL-4 and IL-6 in endometriosis.

Most miRNAs have ability to regulate several hundred transcripts by pairing to 3′-UTR of target genes.^[24]^ In our study, the correlation analysis exhibited that miR-17 was negatively correlated with IL-4 and IL-6. Bioinformatics predicted miR-17 target with 3′-UTR of IL-4 receptor and IL-6 receptor in different positions closely. These results demonstrated downregulated of miR-17 expression may increase receptors of IL-4 and IL-6, and then selected cytokines perform their regulation functions in this disease by combining with receptors. ROC curve analysis revealed the plasma levels of miR-17 was useful biomarker for differentiating women with and without endometriosis, the combination of miR-17, IL-4, and IL-6 could improve the diagnostic power.

Although the results of our study are promising, the sample size was relatively small. The clinical application of large samples is necessary to determine the effectiveness of the potential biomarkers. Besides, we did not factor in the patients’ BMI due to lack of corresponding data. Many research have demonstrated serum levels of IL-6 are increased in overweight and obese subjects.^[25,26]^ Furthermore, endometriosis shows an inverse correlation with BMI and lower BMI is considered as a predictive factor not only for any type of endometriosis but also for severe ones.^[27–29]^ We need longer follow-ups to determine if BMI influence diagnostic performances of miR-17, IL-4, and IL-6.

## 5 Conclusion

The present study characterizes the role of miR-17, IL-4, and IL-6 in the pathogenesis of endometriosis, suggesting the feasibility of using miR-17 and selected cytokines as a noninvasive diagnostic test for the detection of endometriosis.

## Author contributions

**Data curation:** Danting Jin.

**Investigation:** Fang Wang.

**Methodology:** Hongxia Wang.

**Writing – original draft:** Yang Zhang.
